# Supplementary material for: Long-read sequencing identified a causal structural variant in an exome-negative case and enabled preimplantation genetic diagnosis
Source: Hereditas. 2018 Sep 28;155:32. doi: 10.1186/s41065-018-0069-1 (PMC6162922; doi:10.1186/s41065-018-0069-1)

# Additional file 1

Table S1 Characteristics of four embryos after in vitro fertilization. The four embryos were scored according to the Istanbul consensus. Embryo No.1 was transplanted.

| Embryo | Detection results of G6PC gene | | Results of STR loci analysis | Diagnosis result |
| --- | --- | --- | --- | --- |
|  | c.326 G>A | chr17g.41049879_41057003 |  |  |
| No. 1 Grade: 5BB | Heterozygous | NO | The embryo inherited the maternal risk allele, the paternal non-risk allele. | Carrier |
| No. 2 Grade: 5BB | Heterozygous | NO | There are three alleles in D17S760 | Partial trisomy |
| No. 3 Grade: 4BC | Heterozygous | NO | The embryo inherited the maternal risk allele, the paternal non-risk allele. | Carrier |
| No. 4 Grade: 4BB | Heterozygous | NO | The embryo inherited the maternal risk allele, the paternal non-risk allele. | Carrier |

Figure S1. Sanger sequencing on the blood samples (left panel) and sperm samples (right panel) of the father, to determine whether germline mosaicism on c.326G>A is present. However, the results were inconclusive, and indeed a small peak of A allele and an even smaller peak for C allele is present at the c.326 position.


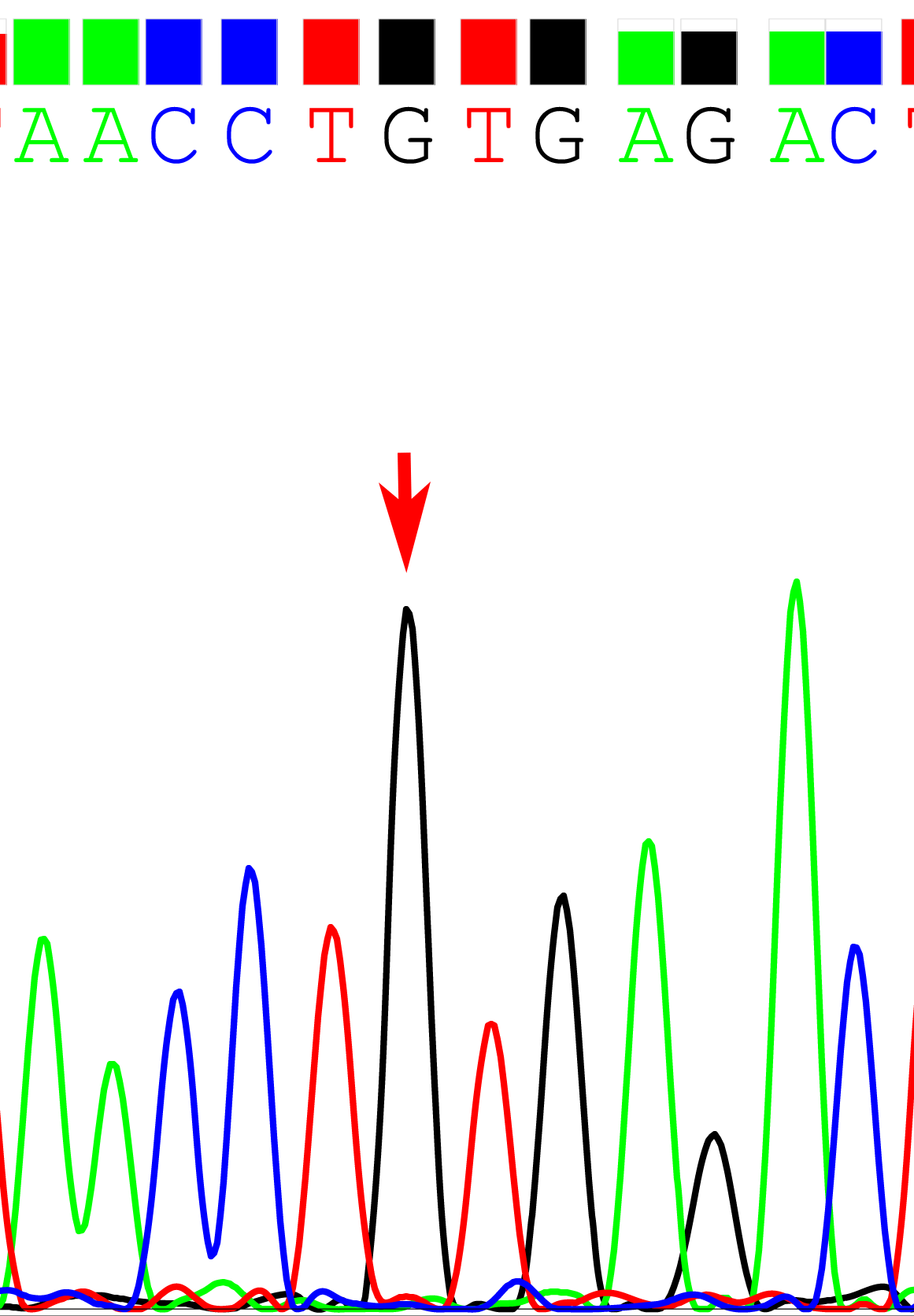

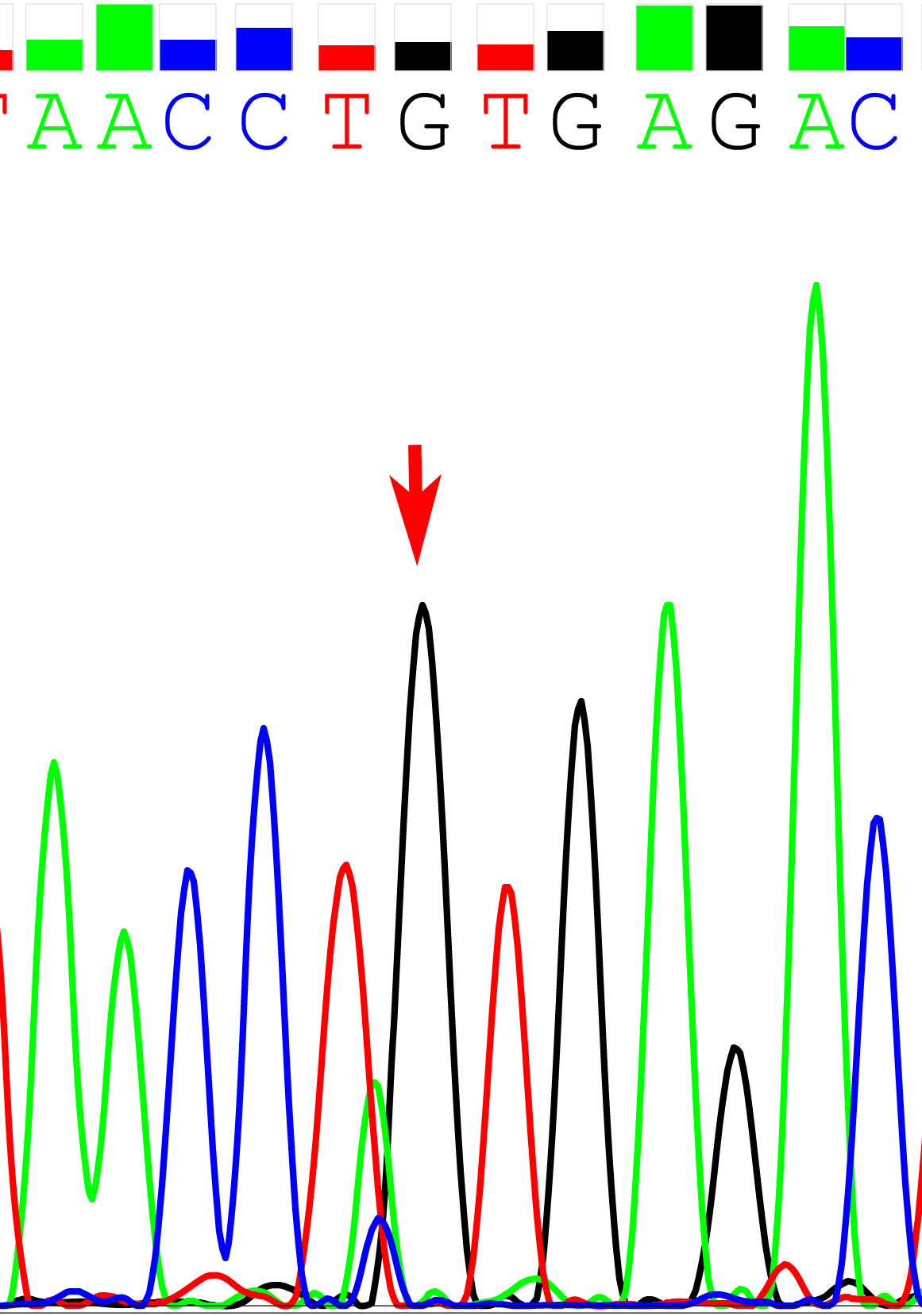


Figure S2. Image of type-B ultrasonic of liver on the newborn baby. B-ultrasonogram showed that the liver and kidneys were normal. SP: Spleen; LK: Left kidney.


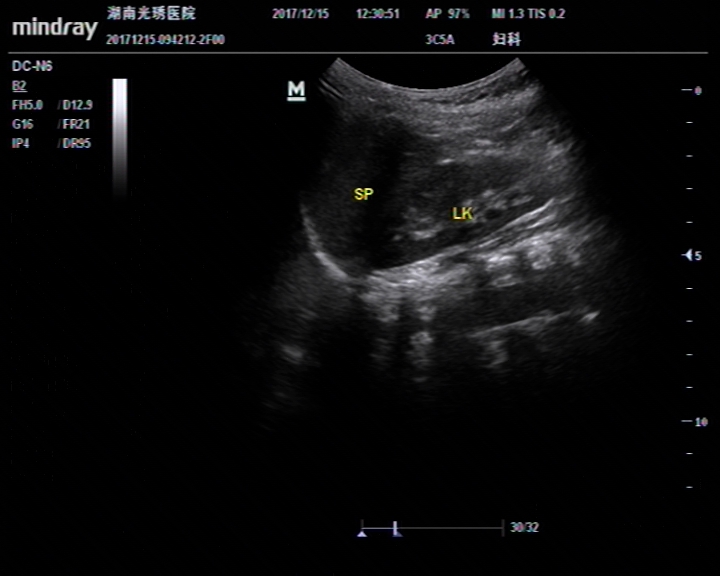

Supplement: Supplementary file 1 — Table S1. Characteristics of four embryos after in vitro fertilization. The four embryos were scored according to the Istanbul consensus. Embryo No.1 was transplanted. Figure S1. Sanger sequencing on the blood samples (left panel) and sperm samples (right panel) of the father, to determine whether germline mosaicism on c.326G > A is present. However, the results were inconclusive, and indeed a small peak of A allele and an even smaller peak for C allele is present at the c.326 position. Figure S2. Image of type-B ultrasonic of liver on the newborn baby. B-ultrasonogram showed that the liver and kidneys were normal. SP: Spleen; LK: Left kidney. (DOCX 372 kb) [file 41065_2018_69_MOESM1_ESM.docx]
